# Expression of T-helper-associated cytokines in patients with type 2 diabetes mellitus with retinopathy

**Published:** 2012-01-28

**Authors:** Hui Chen, Feng Wen, Xiongze Zhang, Shao Bo Su

**Affiliations:** State Key Laboratory of Ophthalmology, Zhongshan Ophthalmic Center, Sun Yat-sen University, Guangzhou, China

## Abstract

**Purpose:**

Recent studies showed that immunological mechanisms were involved in the pathogenesis of diabetic retinopathy (DR). T-helper (Th) cells play an important role in chronic inflammatory disorders and autoimmune diseases. Whether Th cells participate in the pathogenesis of DR remains unclear.

**Methods:**

To evaluate the role of Th cells in the pathogenesis of type 2 diabetes mellitus with retinopathy, the concentrations of interferon (IFN)-γ, interleukin (IL)-1β, IL-2, IL-4, IL-5, IL-6, IL-9, IL-10, IL-12 p70, IL-13, IL-17A, IL-22, and tumor necrosis factor (TNF)-α in the serum of 29 patients with type 2 diabetes mellitus and 30 normal controls were measured with FlowCytomix Technology. IL-22 levels in unstimulated and stimulated peripheral blood mononuclear cells (PBMCs) were examined with enzyme-linked immunosorbent assay.

**Results:**

We found that the mean IL-22 serum levels were slightly lower in diabetic patients than in normal controls. The IL-22 level of PBMCs was significantly elevated in patients with proliferative diabetic retinopathy compared with the level in patients with non-proliferative diabetic retinopathy, patients with non-DR, and healthy controls. Additionally, the IL-22 serum and PBMC levels were positively correlated with the duration of diabetes. Serum levels of other associated cytokines showed no significant change in diabetic patients compared to controls.

**Conclusions:**

These results indicate a possible role of Th22 cells in DR, and IL-22 may be involved more in the pathogenesis of proliferative diabetic retinopathy than in other stages of DR.

## Introduction

Diabetic retinopathy (DR) is a common and progressive complication of diabetes mellitus. DR is characterized by the loss of pericytes, hypertrophy of the basement membrane, microaneurysm formation, increased vascular permeability, capillary occlusions, neovascularization, and fibrovascular proliferation [1]. The etiology and pathogenetic mechanisms of DR have not been clearly elucidated. Recently, immunological mechanisms have been shown to be involved in the pathogenesis of DR [2-4]. According to current data, such as the finding of antipericyte and antiendothelial cell autoantibodies in the circulation of diabetic patients, DR could be considered an autoimmune disease [5]. Additional evidence has implicated autoimmune mechanisms in the proliferative stage of this disease. For example, elevated levels of tumor necrosis factor (TNF)-α, interleukin (IL)-8, and soluble IL-2 receptors were found in the serum of diabetic patients [6]; increased vitreous concentrations of IL-6 and IL-8 were also found in patients with proliferative retinopathy [7].

CD4^+^ T cells are believed to play an important role in the initiation of immune responses. Recent studies have shown that among the T-helper cells, not only Th1/Th2 but also Th17, Th9, and Th22 cells contribute to the pathogenesis of autoimmune diseases. Each subset produces different effector cytokines and attracts different secondary cells into the target organ to induce tissue inflammation. Th1 cells, which predominantly secrete IFN-γ, are thought to play a pathogenic role in organ-specific autoimmune and other chronic inflammatory disorders [8,9]. Th2 cells, which primarily produce IL-4, IL-5, and IL-13, strongly inhibit Th1 differentiation and are responsible for the pathogenesis of allergic diseases [10]. Recently, the identification and cloning of new cytokines have expanded the series of functional subsets of CD4^+^ Th cells. Rather than Th1 cells, CD4^+^ Th cells that produce IL-17, denoted as Th17 cells, have been implicated in the pathogenesis of many chronic inflammatory disorders [11,12]. Moreover, another two subsets of effector CD4^+^ Th cells, denoted as Th9 and Th22 cells, have been described, although their pathological and physiologic roles are still unclear [13-16].

To establish whether Th cells play an important role in the pathogenesis of DR, we investigated the serum levels of Th-associated cytokines in patients with type 2 diabetes mellitus (T2DM) with retinopathy compared with normal controls, and then further investigated the related cytokine level in PBMCs. Moreover, the correlations of several cytokines with disease severity were determined.

## Methods

### Study population

Diabetes was diagnosed according to the 1999 World Health Organization criteria [17]. DR was assessed with fluorescein fundus angiography examination (Zeiss FF450, Jena, Germany). DR was classified according to the new international classification standard for diabetic retinopathy [18]. In the diabetes group, nine had non-DR (NDR), ten had non-proliferative diabetic retinopathy (NPDR), and ten had proliferative diabetic retinopathy (PDR). The exclusion criteria included acute myocardial infarction, organ failure, liver disease, stroke, systemic and recent infection, previous intraocular surgery, earlier intravitreal therapies, photocoagulation in the preceding 3 months, uveitis, trauma, vitreous hemorrhage, and retinal detachment. We also excluded patients who had taken immunosuppressive drugs. Informed consent was obtained from all patients and controls before entering the study. Research was performed in accordance with the ethical standards of the Declaration of Helsinki and the internal Ethics Committees of Sun Yat-sen University, Guangzhou, China, which approved all of the protocols.

### Gender and age breakdown

Patients were recruited from the Department of Ophthalmology of the Zhongshan Ophthalmic Center. All patients with T2DM were receiving appropriate diet and glucose-lowering medicationat the time of recruitment such as insulin, oral hypoglycemic agents, the insulin-sensitizer agent metformin, alone or in combination (insulin treatment [n=9, 31.03%], oral hypoglycemic agents [n=11, 37.93%], metformin [n=20, 68.97%]. We obtained age- and sex-matched samples from 29 T2DM patients and 30 healthy subjects. The mean age of the diabetic subjects (12 men and 17 women) was 55.0 years (SD±10.4 years, range from 31 to 76 years). The mean age of the healthy control subjects (16 men and 14 women) was 57.6 years (SD± 8.3 years, range from 43 to 69 years). Of the 29 T2DM patients, there were nine patients without DR (mean age±SD, 52.4±13.0 years), ten patients with NPDR (58.4±11.4 years), and ten patients with PDR (54.2±5.1 years). The mean hemoglobin A1c (HbA1c) of the diabetic subjects was 7.87±1.01% compared to 5.29±0.50% in the control subjects (normal range HbA1c: 4.27%–6.07%; p<0.05).

### Serum

Five milliliters of whole-blood samples were collected from the patients and healthy controls’ first visit, using a standard venipuncture technique between 9:00 and 11:00 AM and were thawed at room temperature. Serum samples were obtained after centrifugation at 800× g for 10 min, aliquoted, and stored at –80 °C until assayed. The concentrations of IFN-γ, IL-1β, IL-2, IL-4, IL-5, IL-6, IL-9, IL-10, IL-12 p70, IL-13, IL-17A, IL-22, and TNF-α in the sera were determined with specific FlowCytomix kits.

### Cell isolation and culture

The PBMCs were prepared from heparinized blood with Ficoll-Hypaque density-gradient centrifugation. To study the production of related cytokines selected with Multiplex bead analysis of serum, PBMCs were stimulated with phytohaemagglutinin (PHA) at a density of 2×10^6^ cells/ml for 48 h and subsequently used for analysis with enzyme-linked immunosorbent assay (ELISA).

### Immunoassays to quantify cytokines with FlowCytomix kits

The sera were assayed to determine the concentrations of secreted cytokines with FlowCytomix kits (Bender MedSystems, Vienna, Austria) according to the manufacturer’s instructions. These kits allowed the simultaneous quantification of 13 cytokines, including Th1 cytokines (IFN-γ, IL-2, and TNF-α), Th2 cytokines (IL-10, IL-4, IL-5, IL-6 and IL-13), Th17 cytokines (IL-17 and IL-23), Th9 cytokines (IL-9), and Th22 cytokines (IL-12, IL-22). In brief, FlowCytomix technology is based on spectrally discrete microspheres used as the solid phase in an immunoassay. The beads are internally dyed with Starfire Red, a far-red (685- to 690-nm)-emitting fluorochrome excited by ultraviolet, argon, or helium–neon lasers. The test samples were analyzed with flow cytometry using Coulter Epics FC500 (Beckman Coulter, Inc., Fullerton, CA). For each analysis, up to 10,000 events were acquired. The mean concentration of each cytokine was expressed as pg/ml. Using these kits, the minimum detectable concentrations of IFN-γ, IL-1β, IL-2, IL-4, IL-5, IL-6, IL-9, IL-10, IL-12 p70, IL-13, IL-17A, IL-22, and TNF-α were 1.6 pg/ml, 4.2 pg/ml, 16.4 pg/ml, 20.8 pg/ml, 1.6 pg/ml, 1.2 pg/ml, 1.5 pg/ml, 1.9 pg/ml, 1.5 pg/ml, 4.5 pg/ml, 2.5 pg/ml, and 43.3 pg/ml, respectively.

### Enzyme-linked immunosorbent assay for IL-22 in peripheral blood mononuclear cells

The protein levels of IL-22 in the collected supernatants were measured by using a Duoset ELISA development kit (R&D Systems, Minneapolis, MN). The minimal detectable concentration was 20 pg/ml. All samples were measured in duplicate.

### Statistical analysis

Data were analyzed using the statistical package for social sciences (SPSS Version 19.0 for Windows). Group differences between samples from diabetics and controls were analyzed using the two-tailed Student *t* test or two-tailed Mann–Whitney test depending on normality assumptions and the homogeneity of variances. The differences in biochemical parameters among the groups with different degrees of DR severity were evaluated with one-way ANOVA with a post-hoc Scheffe test. Graphs were prepared with Prism version 5 (GraphPad Software Inc., La Jolla, CA). For all tests, p values less than 0.05 were considered significantly different.

## Results

### Patients’ clinical characteristics

The clinical characteristics of the enrolled patients are shown in Table 1. With regard to age, sex, and body mass index (BMI) distribution, no significant difference was observed between the study groups (p=0.363, p=0.232, p=0.536, respectively). The mean duration of diabetes was significantly longer in the PDR group than in the NPDR and NDR groups (p<0.01), although no significant difference was found between the NPDR and NDR groups (p=0.509).

**Table 1:** Clinical and biochemical characteristics of type 2 diabetic patients and healthy control subjects.

**Parameters**	**PDR (n=10)**	**NPDR (n=10)**	**NDR (n=9)**	**Control (n=30)**	**p**
Sex (m/f)	4/6	2/8	5/4	16/14	0.278
Age (years)	54.2±5.1	58.4±11.4	52.4±13.0	57.6±8.3	0.363
BMI (kg/m2)	24.13±3.43	22.9±4.17	22.32±2.13	22.92±2.21	0.536
Diabetes duration (years)	15.2±4.4	7.8±2.5	5.8±2.0	-	<0.001*
FPG (mmol/l)	10.28±2.22	9.34±1.93	7.8±0.85	-	0.014*
HbAlc (%)	8.41±0.93	7.94±1.07	7.27±0.76	-	0.035*

DR: type 2 diabetic patients with retinopathy; NDR: type 2 diabetic patients without retinopathy; NPDR: non-proliferative retinopathy; PDR: proliferative diabetic retinopathy; BMI, Body mass index; FPG: fasting plasma glucose; HbA1c, glycated hemoglobin Data are expressed as mean±SD * p≤0.05.

HbA1c values between 4.27% and 6.07% were considered normal in our laboratory. HbA1c and fasting glucose levels were also found to differ significantly between the NDR and PDR groups (p<0.001, p=0.003, respectively).

### Expression profiles of cytokines in the serum

**Figure 1:**
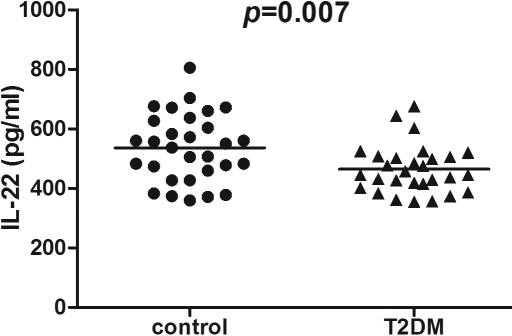
Interleukin (IL)-22 levels in the serum from type 2 diabetes mellitus (T2DM) patients (n=29) and healthy control subjects (n=30) were measured by FlowCytomix. Student's *t*-test showed that concentrations of IL-22 in serum were significantly lower in patients with T2DM than in healthy control subjects (p=0.007).

**Figure 2:**
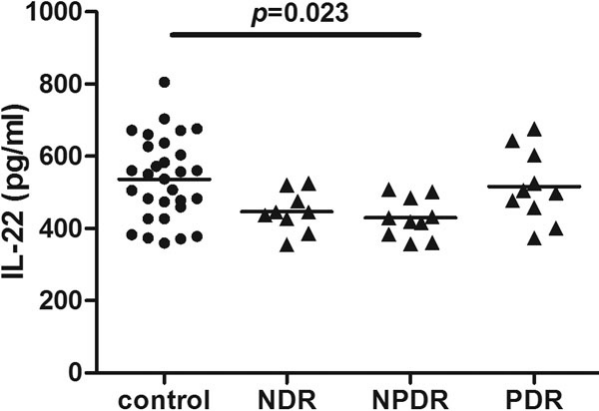
Distribution of serum interleukin (IL)-22 concentrations in controls (n=30), non-diabetic retinopathy (NDR, n=9), non-proliferative diabetic retinopathy (NPDR, n=10) and proliferative diabetic retinopathy (PDR, n=10) were measured by FlowCytomix. One-way ANOVA with a post-hoc Scheffe test showed that the serum level of IL-22 was significantly lower in NPDR compared with the controls (p=0.023). No significant difference was found among NDR, NPDR, and PDR groups.

After adjusting for age, sex, and BMI, we observed that the IL-22 serum level was significantly decreased in the diabetic group compared to the controls (p=0.007) (Figure 1). However, the concentration of IL-22 in serum was not significantly different among the NDR, NPDR, and PDR groups (Figure 2). The mean serum levels of IL-1β, IL-2, IL-5, IL-6, IL-9, IL-10, IL-13, IL-17A, and TNF-α demonstrated no significant difference in patients compared to the controls (Table 2). IFN-γ and IL-12 serum levels were below the detection limits. The serum levels of all investigated parameters did not correlate with the DR stage (p>0.05).

**Table 2:** IL-1β, IL-2, IL-5, IL-6, IL-10, IL-13, IL-17A, IL-22, TNF-α in the serum samples of T2DM and control patients (pg/ml).

**Cytokines studied**	**T2DM (n=29)**	**Control subjects (n=30)**	**p**
IL-1β	3.82±3.0	8.35±6.20	0.113
IL-2	17.10±13.08	13.12±10.45	0.636
IL-5	8.01±5.3	6.45±3.10	0.361
IL-6	16.99±33.93	25.27±21.29	0.442
IL-9	4.4	3.47±0.64	0.185
IL-10	2.68±2.23	3.08±1.91	0.603
IL-13	12.32	19.79±4.86	0.263
IL-17A	3.37±2.0	3.59±0.33	0.836
IL-22	464.51±80.62	536.16±114.26	0.007*
TNF-α	28.88±29.85	38.56±51.26	0.508

Controls: healthy subjects; T2DM: type 2 diabetes mellitus patients; IL, interleukin; TNF: tumor necrosis factor Data are expressed as mean±SD * p≤0.05.

### Concentrations of IL-22 in the supernatants of cultured peripheral blood mononuclear cells

**Figure 3:**
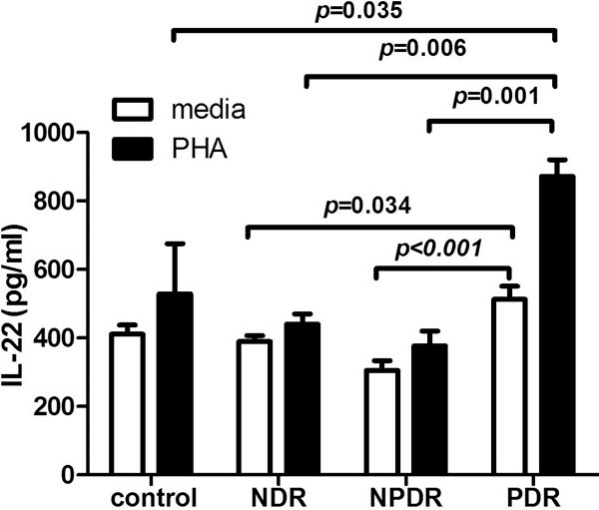
Interleukin (IL)-22 in the supernatants of peripheral blood mononuclear cells (PBMCs) from NDR (n=9), NPDR (n=10), PDR (n=10) and normal control subjects (n=10) were measured by ELISA. Separated PBMCs were cultured with or without Phytohaemagglutinin (PHA, 0.02%) for 48 h. Data are represented as means±SD. One-way ANOVA with a post-hoc Scheffe test demonstrated a significantly higher expression in patients with PDR than in patients with NPDR (p<0.001) and NDR (p=0.034). IL-22 production was significantly increased after PHA stimulation, with a higher upregulation in patients with PDR than in patients with NPDR (p=0.001), NDR (p=0.006) and control subjects (p=0.035).

The results showed that significantly higher expression was noted in patients with PDR (n=10) than in patients with NPDR (n=10; p<0.001) and NDR (n=9; p=0.034). Following PHA stimulation, IL-22 production was significantly increased, with a higher upregulation in patients with PDR than in patients with NPDR (p=0.001), patients with NDR (p=0.006), and normal control subjects (n=10, p=0.035) (Table 3, Figure 3).

**Table 3:** Concentrations of IL-22 in culture supernatants of unstimulated or activated PBMCs from type 2 diabetes mellitus and healthy controls.

**Cytokine**	**PBMCs**	**Control subjects (n=10)**	**NDR (n=9)**	**NPDR (n=10)**	**PDR (n=10)**	**p**
IL-22	(unstimulated)	411.25±85.16	389.69±52.73	305.36± 89.73	513.08±119.97	<0.001
	(stimulated)	528.73± 463.64	440.50±89.47	377.01± 138.32	871.72±153.00	0.001

NDR, non diabetic retinopathy; DR, diabetic retinopathy; IL, interleukin; IFN-γ, interferon-gamma, PBMCs, peripheral blood mononuclear cells. All cytokines expressed as pg/ml. Data are means±SD.

### Correlation analyses in diabetic retinopathy

**Figure 4:**
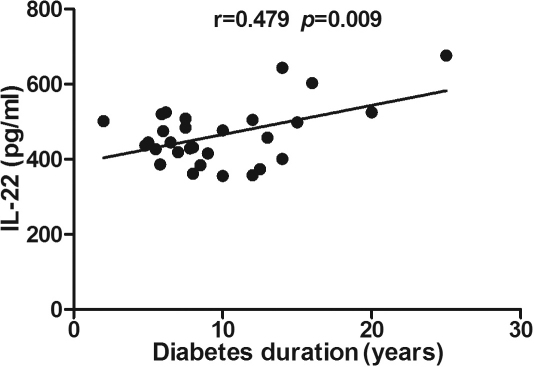
Correlation analysis showed that interleukin (IL)-22 concentrations in serum were positively correlated with the duration of diabetes(r=0.479, p=0.009). Pearson’s correlation test was used (p<0.05 significance; r=correlation coefficient).

**Figure 5:**
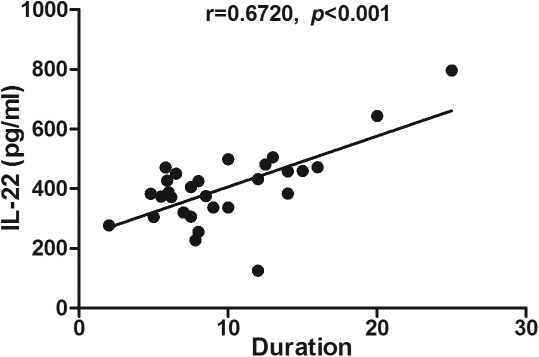
Correlation analysis showed that interleukin (IL)-22 concentrations in unstimulated peripheral blood mononuclear cells (PBMCs) were positively correlated with the duration of diabetes (r=0.672, p<0.001) .Pearson’s correlation test was used (p<0.05 significance; r=correlation coefficient).

**Figure 6:**
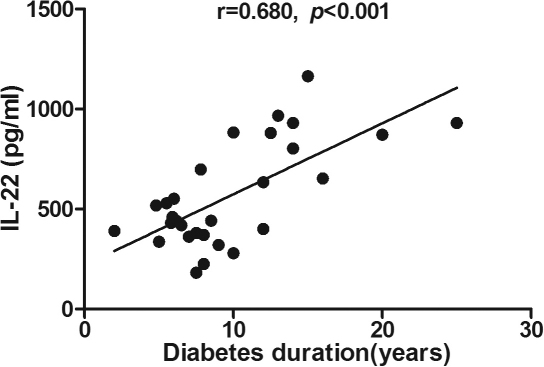
Correlation analysis showed that interleukin (IL)-22 concentrations in stimulated peripheral blood mononuclear cells (PBMCs) were positively correlated with the duration of diabetes(r=0.680, p<0.001). Pearson’s correlation test was used (p<0.05 significance; r=correlation coefficient).

Correlation analysis showed that IL-22 concentrations in serum (r=0.479, p=0.009), unstimulated (r=0.672, p<0.001) and stimulated PBMCs (r=0.680, p<0.001) were positively correlated with the duration of diabetes (Table 4, Figure 4, Figure 5, and Figure 6). No other significant correlations were found among the cytokine levels analyzed. No correlation was demonstrated between serum cytokine concentrations and HbA1c or fasting plasma glucose (FPG) levels.

**Table 4:** Correlation between IL-22 and age, gender, BMI, duration of diabetes and HbA1c levels in T2DM.

**Variables**	**Serum**		**Unstimulated PBMCs**		**Stimulated PBMCs**	
	**Coefficient**	**p**	**Coefficient**	**p**	**Coefficient**	**p**
Age	−0.25	0.191	−0.157	0.341	0.149	0.366
Gender	0.129	0.504	−0.166	0.311	0.212	0.194
BMI	0.356	0.058	0.155	0.346	0.072	0.663
Duration of T2DM	0.479	0.009*	0.672	<0.001*	0.68	<0.001*
FPG	0.197	0.305	0.078	0.686	0.122	0.529
HbAlc	0.061	0.754	0.279	0.143	0.256	0.18

T2DM, type 2 diabetes mellitus; BMI, Body mass index; FPG: fasting plasma glucose; HbA1c, glycated hemoglobin; IL, interleukin. The coefficients were calculated from the Pearson correlation test * p≤0.05.

## Discussion

Th cells play a critical role in the pathogenesis of chronic inflammation disease. However, whether this mechanism happens in patients with diabetes is currently unknown. Th22 is a new subset of T cells clearly distinct from Th17, with distinct gene expression and function. IL-22 is expressed by Th22 and highly upregulated during chronic inflammatory diseases [19,20]. The present study indicated that IL-22 was slightly decreased in the T2DM serum. However, is this small decrease of clinical relevance?

IL-22, a member of the IL-10 family of cytokines, is produced by special immune cell populations, including Th22, Th1, and Th17 cells. Additionally, IL-22 has been most commonly considered a proinflammatory cytokine due to its expression in the lesions of patients with chronic inflammatory diseases, such as rheumatoid arthritis, Crohn’s disease, psoriasis, and atopic dermatitis by inducing other proinflammatory cytokines, such as IL-6, IL-8, and TNF-α [21]. However, IL-22 has also shown a less direct inflammatory role and instead induced the expression of genes associated with antimicrobial defense and cellular differentiation [22,23]. Previous studies have shown decreased serum and plasma levels of IL-22 in patients with sarcoidosis and systemic lupus erythematosus [24-26]. Moreover, Sugimoto et al. [27] reported that IL-22 ameliorated intestinal inflammation in a mouse model of ulcerative colitis. Evidence has shown that IL-22 contributed to regulating hepatitis and is protective in inflammatory bowel disease [28,29]. The cause of this IL-22 disparity remains elusive. It could be explained by the theory that IL-22 plays a dual role in inflammation depending on the specific tissue [27]. Apart from IL-22’s dual role in autoimmune disease, what degree of under-expression of IL-22 is detrimental for the eye is currently unknown. Hence, the small change in the IL-22 level in serum might exert little influence on T2DM.

In addition, the serum concentrations of cytokines are influenced by many factors, and several types of cells could produce cytokines. Thus, we further specifically used PBMC cultures to evaluate IL-22 levels in patients and controls. Our results demonstrated that the levels of IL-22 were significantly increased in the PBMCs of patients with PDR compared with the PBMCs of patients with NPDR, patients with NDR, and healthy controls, implying that Th22 may be involved in the pathogenesis of PDR.

PDR is the advanced stage of DR characterized by uncontrolled vascular proliferation leading to fibrosis and traction retinal detachments [30]. As in many fibrotic eye diseases such as age-related macular degeneration, an injury to the retina could result in inflammatory changes, tissue edema, and hypoxia-driven neovascularization prone to hemorrhage, leading to further activation of the wound-healing response, and ultimately development of severe fibrosis [31]. In addition to inflammation induced by autoimmune or infectious diseases, IL-22 also plays a role in fibrosis after wounding. IL-22 is highly expressed in rheumatoid arthritis (RA), in which IL-22 induces production of monocyte chemoattractant protein-1 (CCL2; also known as MCP-1) and proliferation of synovial fibroblasts and osteoclasts [32,33]. Raised vitreous CCL2 levels have been reported in several studies. A possible role for CCL2 in PDR was suggested in an vitro wound-healing model [34,35]. Recently, several fibroblast growth factors, such as connective tissue growth factor (CTGF) levels, were found to correlate significantly with the degree of fibrosis in various vitreoretinal disorders including proliferative vitreoretinopathy and PDR [36,37]. Th22 cells also produce fibroblast growth factors [38]. Therefore, Th22 cells may be involved in the pathogenesis of PDR by regulating angiogenesis and fibrosis. On the other hand, IL-22 has minor proinflammatory effects, and in some cases such as uveitis, IL-22 seems to play an aggravating role in the pathogenesis of autoimmune disease. IL-22 is expressed in uveitis, in which IL-22 decreases total tissue resistance and induces apoptosis in retinal pigment epithelial cells [39]. Therefore, we speculate that the Th22 cells of peripheral blood may reach the eye and play some pathophysiologic role in this way. Because IL-22 exhibits potent angiogenic or angiostatic activity, targeting IL-22 might offer a unique approach to regulate angiogenesis and fibrosis. However, the clinical association between Th22 cells and PDR remains to be elucidated.

Another interesting finding in this study was that IL-22 exhibited a positive correlation with the duration of diabetes. However, the duration of diabetes may not accurately reflect a patient’s true history because diabetes can often go undetected for several years. No association of serum IL-22 levels with the severities of DR was found in our study, which may be explained by the relatively small sample size. Therefore, prospective cohort studies with larger sample sizes are needed.

In our analyses of serum cytokine profiles, no cytokine differed significantly between T2DM patients and controls, except IL-22, whereas levels of these cytokines were increased or undetectable in patients with type 2 diabetes. Various investigators have detected elevated levels of IL-6 in ocular fluid from patients with diabetic retinopathy [40]. Serum concentrations of TNF-α were reported to be elevated in diabetic patients [41]. Moreover, in a recent study, non-inflammatory cytokines, such as IL-2, IL-4, IL-10, and a major proinflammatory cytokine (IFN-γ) were not detected in vitreous fluid in patients with diabetic retinopathy [42]. Therefore, to clarify the role of Th cells in T2DM patients with retinopathy, further studies on multiple potential origins, such as peripheral blood mononuclear cells or vitreous fluid, are required.

In conclusion, an increase in IL-22 levels in patients with PDR suggests that IL-22 may be associated with the pathogenetic mechanism of this disease and could be an important target for developing new drugs and treatments for PDR.
